# CLAVATA3 Dodecapeptide Modified CdTe Nanoparticles: A Biocompatible Quantum Dot Probe for In Vivo Labeling of Plant Stem Cells

**DOI:** 10.1371/journal.pone.0089241

**Published:** 2014-02-24

**Authors:** Guanghui Yu, Yanping Tan, Xiangzhu He, Yonghua Qin, Jiangong Liang

**Affiliations:** 1 Key Laboratory for Biotechnology of the State Ethnic Affairs Commission, College of Life Sciences, Hubei provincial Key laboratory for protection and application of special plants in Wuling Area of China, South-Central University for Nationalities, Wuhan, Hubei, China; 2 College of Electronics and Information Engineering, South-Central University for Natonalities, Wuhan, Hubei, China; 3 College of Science, State Key Laboratory of Agricultural Microbiology, Institute of Chemical Biology, Huazhong Agricultural University, Wuhan, Hubei, China; University of Helsinki, Finland

## Abstract

CLAVATA3 (CLV3) dodecapeptides function in plant stem cell maintenance, but CLV3 function in cell-cell communication remains less clear. Here, we coupled CLV3 dodecapeptides to synthesized CdTe nanoparticles to track their bioactivity on stem cells in the root apical meristem. To achieve this, we first synthesized CdTe quantum dots (QDs) using a one-pot method, and then evaluated the cytotoxicity of the QDs in BY-2 cells. The results showed that QDs in plant cells must be used at low concentrations and for short treatment time. To make biocompatible probes to track stem cell fate, we conjugated CLV3 dodecapeptides to the QDs by the zero-coupling method; this modification greatly reduced the cytotoxicity of the QDs. Furthermore, we detected CLV3-QDs localized on the cell membrane, consistent with the known localization of CLV3. Our results indicate that using surface-modified QDs at low concentrations and for short time treatment can improve their utility for plant cell imaging.

## Introduction

**Figure 1:**
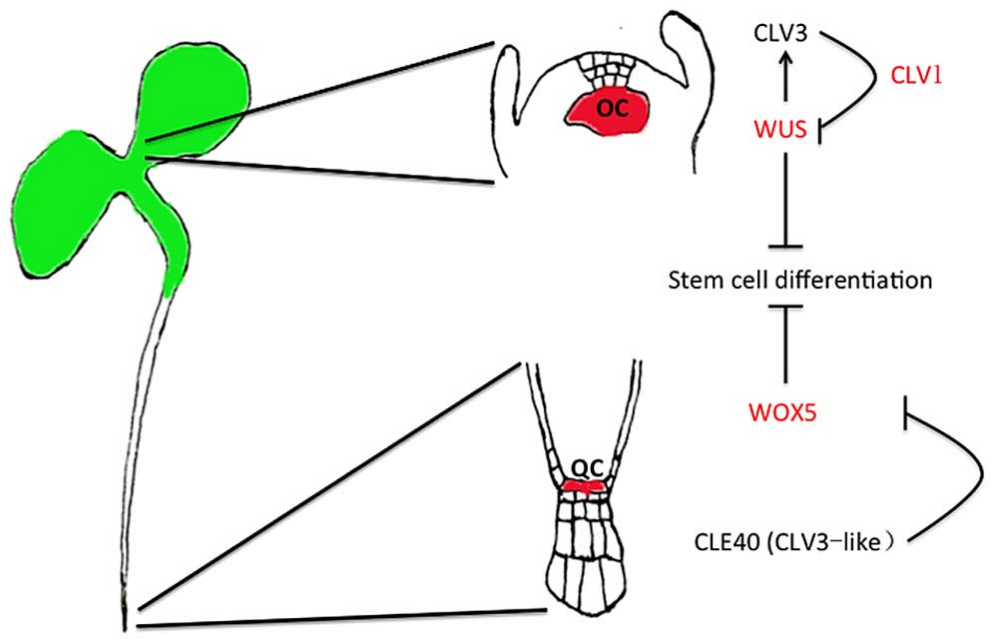
Schematic of stem cell niche organization and maintenance in the plant shoot apical meristem (SAM) and root apical meristem (RAM). This conserved pathway involves feedback regulation of transcription by CLE dodecapeptides. The organizing center (OC) in SAM stem cell niche and the quiescent center (QC) in RAM stem cell niche are indicated in red in the diagram (Adapted from [3]).

In plant postembryonic development, continuous growth stems from the meristematic cells, which maintain a dynamic balance between cell division and differentiation [1]. In the model plant Arabidopsis (*Arabidopsis thaliana*), the aboveground tissues originally come from the shoot apical meristem (SAM), which initiates as a vegetative meristem making leaves. The SAM later changes into an inflorescence meristem, which bears determinate floral meristems producing reproductive organs. Underground tissues derived from the root apical meristem (RAM) absorb nutrients from the extracellular environment. Stem cells are found in the organizing center in the SAM, and the quiescent center in the RAM (Figure 1). Complex cell-to-cell communication maintains the balance between stem cell division and differentiation; this dynamic balance within the stem cell niche and surrounding cells plays a crucial role in plant development [1].

A well-studied pathway for stem cell maintenance involves CLAVATA3 (CLV3), one of 32 members of the large CLE peptide family. Some CLE family members, for example CLV3 in the SAM, and CLE40 in the RAM, cause termination of meristem activity by inducing terminal differentiation of stem cells [1]. CLV3, a 12–14 amino acid peptide, coordinates cell proliferation and differentiation in the plant stem cell niche [2]. In this signaling pathway, peptides secreted from the organizing center (OC) and the quiescent center (QC) cells determine the fate of the surrounding stem cells. CLE regulates stem cell fates by binding CLV family kinase receptors, e.g. CLV1, or/and CLV2, to regulate the down-stream regulator WUSCHEL (WUS). Meanwhile, CLV3 also regulates the expression of *WUS* (in the OC) and *WOX5* (in the QC) to maintain cell numbers in the stem cell niche [3] (Figure 1). Chemically synthesized CLV3 has a similar effect to CLE40 on root growth, depleting stem cell populations [1]. Although the function of specific CLE peptides in plants, in most cases, remains unclear [4], [5], application of synthetic bioactive CLE peptides in roots has proven useful to decipher CLE signaling. In this study, we used nano-technology to link CLV3 dodecapeptides to quantum dots (QDs) to make fluorescent probes for tracking CLE ligand-receptor binding, exploring the use of this new technology to study peptide signaling in plant stem cell maintenance.

In the past decades, fluorescent quantum dots (QDs) have had a substantial impact on biological and medical technology in cell labeling and cell imaging [6]–[8]. The distinctive advantages of QDs compared to conventional organic dyes, particularly their optical and electronic properties, have motivated scientists to dope or modify the surface of QDs to make biocompatible or bioactive fluorescent probes for bimolecular tracking or living cell labeling. However, several factors, including size and stability, restrict the utility of functionalized biocompatible QDs with specific targeting and excellent fluorescence properties. For example, smaller QDs generally prove more suitable for biological applications than larger QDs [8]. However, the smaller size also leads to QD instability and a reduction in fluorescence quantum yield [8]. For biological imaging, one popular strategy is to conjugate reactive biomolecules, such as enzymes, antibodies, nucleic acids or polyethylene glycol to the QD surface, to promote specific interactions with biological targets [9]. Although this is often necessary to minimize nonspecific interactions of the QDs with biological material, these modifications inevitably increase the size of the QDs, probably perturbing the behavior of the labeled molecules and impairing their ability to access small spaces, such as the cleft at excitatory neuronal synapses [10]. For example, compared directly with small dyes, QD modification slowed down membrane diffusion of glutamate receptors [11] and changed the type of motion of potassium channels [12]. Therefore, developing new surface coatings and methods to reduce QD size will improve their utility. Reducing the thickness of the ZnS shell or directly synthesizing QDs with a more stable core, such as CdTe may reduce QD size. However, although the protocol to synthesize CdTe QDs is relatively mature [13], [14], applications in cell imaging require the assessment of QD cytotoxicity. Most research uses animal cells for toxicity assessment and QD labeling [15], [16]; only a few studies have directly examined the effect of QDs on plant cells. Moreover, the cell wall obstructs the application of QDs in plant cells. Herein, as a preparation to track plant stem cell fate by using QDs modified with CLV3 dodecapeptides, we optimized the application condition of CdTe QDs in reducing or eliminating the toxicity QDs to plant cells of the BY-2 cell line.

## Materials and Methods

### Reagents

All reagents were purchased from Sigma-Aldrich (Shanghai, China) unless otherwise stated.

### Synthesis of CdTe QDs

Water-soluble CdTe QDs were synthesized according to the published method, with slight modifications [17]. Briefly, 10 ml of 10 mM CdCl_2_ and 38 ml of ultrapure water were transferred to a small flask. The solution was mixed with 10 µl of thioglycolic acid (TGA) and kept bubbling with high-purity N_2_. 1.0 M NaOH was added to adjust the pH to 11.0, and the mixture became clear. Then 53.8 mg trisodium citrate and 2.0 ml of 10 mM Na_2_TeO_3_ were injected into the mixture. Finally, 3.0 mg NaBH_4_ was added under N_2_ atmosphere. After mixing, this solution was transferred to a reaction kettle and kept at 100°C to produce water-soluble CdTe QDs. The reaction time is 1–2 h. The resulting products were precipitated by acetone, and superfluous reactants were removed by centrifugation at 10,000 rpm for 3 min. The precipitate was re-dispersed in water, and stored at 4°C. The QD size and concentration was calculated by the method previously reported [18].

### Cell culture

Suspension-cultured cells of tobacco (*Nicotiana tabacum* L.) cv. BY-2 were used for the cytotoxicity assay. The culture medium was the standard MS (Murashige and Skoog) medium supplemented with 3% sucrose and 2 mg L^−1^ 2,4-D (2,4-dichlorophenoxyacetic acid) (pH 5.8) [19]. The culture was incubated on a rotary shaker at 100 rpm and at 25°C in the dark for 2 d or 4 d. Four-d-old BY-2 cells cultured in the media were used for experiments. The Cd^2+^ concentration at 1.0 µM was set as the control as previously reported [20].

### Membrane permeabilization assay

The fluorescent dye SYTOX Green (catalog number S7020, Invitrogen Molecular probes, 5.0 mM solution in DMSO) was used for the membrane permeabilization assay according to a previous report [21]. An aliquot of BY-2 suspension cells was incubated with 0.025% (w/v) SYTOX Green at room temperature for 1 h and subsequently washed with culture solution. The stained cells were observed and photographed by fluorescence microscopy.

### Determination of intracellular ROS

A procedure to detect general ROS production [22] was used in BY-2 cell assays. An aliquot of BY-2 suspension cells was incubated in growth medium containing carboxy-H_2_DCFDA [5-(and-6)-carboxy-2′, 7′-dichloro-dihydrofluorescein diacetate] (catalog number C400, Invitrogen Molecular probes, 50 mM stock solution in DMSO; final concentration 2.0 µM) for 1 min at room temperature before images were captured with a single 500 ms exposure. Microscopy was performed with Nikon C1-Si Confocal laser-scanning microscope fitted with CCD. A 450- to 490-nm excitation filter and a 515-nm emission filter were used. Image-capture and analysis was performed using EZ-C1 software.

### Cell death assay

Cell death assays by Evans Blue staining were conducted based on a published protocol [23]. An aliquot of BY-2 suspension cells was incubated with 0.025% (w/v) Evans Blue at room temperature for 10 min and subsequently washed with 100 mM CaCl_2_ (pH 5.6). The stained cells were observed and photographed by light microscopy. Evans Blue-positive cells were counted using Image J software. All measurements were done in three or more independent biological experiments.

### Real-time PCR analysis

To analyze mRNA expression of genes involved in Cd^2+^ responses, cells were incubated as above for the indicated time, and then total RNA was isolated using the TRIzol reagent (catalog number 15596-026, Invitrogen). The quantity and purity of RNA was verified by measuring the wavelength of A260 and A280. cDNA was synthesized from total RNA (2.0 µg) with oligo (dT)18 primers (0.5 µg) using Prime RT Premix (Toyobo, Japan) according to the manufacturer's instructions. cDNA was added to FastStart Universal SYBR Green Master (Rox) (Roche Applied Sciences) and subjected to quantitative real-time PCR analysis using Rotor-Gene 3000 (Corbett Life Science). The primers used in this study were as follows: forward primer of Phytochelatin synthase 1 (*PCS1*) (AY235426.1) is 5′-TGGACTGTTGTGAGCCTCTG-3′, reverse primer is 5′-AGGCCATGACTTGTTTACGG-3′; Forward primer of *Actin* (AB158612) is 5′-AAGGTTACGCCCTTCCTCAT-3′, reverse primer is 5′-CATCTGTTGGAAGGTGCTGA-3′. *Actin* was used as an internal control. The PCR conditions were 10 min denaturation at 95°C, followed by 45 cycles at 95°C for 30 s, 55°C for 30 s, and 72°C for 30 s (*PCS1*) and 94°C/1 min; 94°C/30 s, 59°C/30 s, 72°C/1 min, 30 cycles (*Actin*), respectively, and 72°C/10 min for extension. To verify that only the specific product was amplified, a melting point analysis was performed after the last cycle by cooling samples to 55°C and then increasing the temperature to 95°C at 0.2°C/s. Results were calculated using the ΔCt method normalizing to *Actin* expression for each sample.

### Peptide synthesis, quantum dot conjugation and mobility shift assay

The dodecapeptide (NH_2_-RTVPSGPDPLHH-COOH) was synthesized by Fmoc (9-fluorenylmethyloxy-carbonyl) solid-phase peptide synthesis using a peptide synthesizer (ABI model 433A). The peptides were purified by reverse-phase HPLC comprising two solvent delivery pumps and a UV detector set at 220 nm. The column used was a Develosil-ODS HG-5, 10 mm i.d. ×250 mm, coupled to a guard column (Nomura Chemical Co.). The separation was carried out with a linear gradient of solvent A (H_2_O/CH_3_CN 98∶2+0.5% acetic acid) into solvent B (H_2_O/CH_3_CN 30∶70+0.5% acetic acid): 0–50% B over 20 min, 50–100% B over 5 min, run at 3.0 ml min^−1^. The purity of each peptide was assessed by monitoring its UV absorbance (220 nm) and total ion chromatogram of liquid chromatography-mass spectrometry (LC-MS) analysis.

The immobilization of peptides on the surface of QDs was accomplished by using a modified version of previously reported methods [24], [25]: 5.0 mg EDC (catalog number 77149, Thermo scientific), 3.75 mg sulfo-NHS (catalog number 24510,Thermo scientific), 0.2 ml 2-Morpholinoethanesulfonic acid (MES) buffer (0.1 M MES, pH 6.0), and 0.3 ml distilled water were added to 0.5 ml carboxyl-CdTe QDs (10^−5^ M), stirred and allowed to react for 30 min. Next, the QDs were centrifuged at 4000 r/min for 40 min to remove extra chemicals. The pellets were then gently rinsed twice with 1.0 ml double-distilled water (DDI). The following were then added to the precipitate: 0.5 ml peptide solution (1.0 mg ml^−1^), 0.3 ml DDI water, and 0.2 ml sodium borate buffer (pH 8.3 0.1 M boric acid). This solution was then incubated in an ultrasonic bath until the pellet dissolved. After waiting for 2 hours at room temperature to allow the reaction to take place, the solution was stored at 4°C overnight. The excess peptides and reaction by products were removed by several rounds of centrifugation.

Gel electrophoresis was performed according to the procedure of Pinaud et al [26], which was conducted on 0.5% agarose gels in TBE buffer (89 mM Tris, 89 mM borate and 2.0 mM EDTA, pH 8.3) for 10 min at 120 V. The gel bands were detected on a gel scanner (Bio-Rad, Gel 2000) with UV light excitation and were transformed to fluorescence bands by Image J software.

### Protoplast preparation and cell labeling

Protoplasts were isolated from 7-day-old *Arabidopsis* roots according to a previously reported method [27] with minor modifications. Briefly, segments of roots were immersed in a petri dish containing 2 ml enzyme solution [1/2 MS, 3% sucrose, 0.4 M glucose, 2.5% cellulase (Onozuka R-10), 1% pectinase (Merck reagent), 1% macerozyme R-10 (Serva), pH 5.8] and digested overnight. The enzyme solution (supernatant) was removed and protoplasts were washed with protoplast medium (1/2 MS, 3% sucrose, 0.4 M glucose, pH 5.8). Samples were spun for 5–10 minutes at 50 ×g to remove supernatant. The wash was repeated, and then the protoplasts were resuspended in protoplast medium for further cell labeling. After 3–4 hours incubation of the QD-peptide probe with the protoplasts, the cells were carefully washed 3 times in the medium with a micropipette and observed by confocal microscopy.

### Statistical analysis

Data were analyzed by using SPSS 20.0. Statistical significance was determined by a one-way ANOVA combined with post-hoc analysis. Differences were considered significant for *P*<0.05.

## Results

### Characterization of TGA-capped CdTe QDs

**Figure 2:**
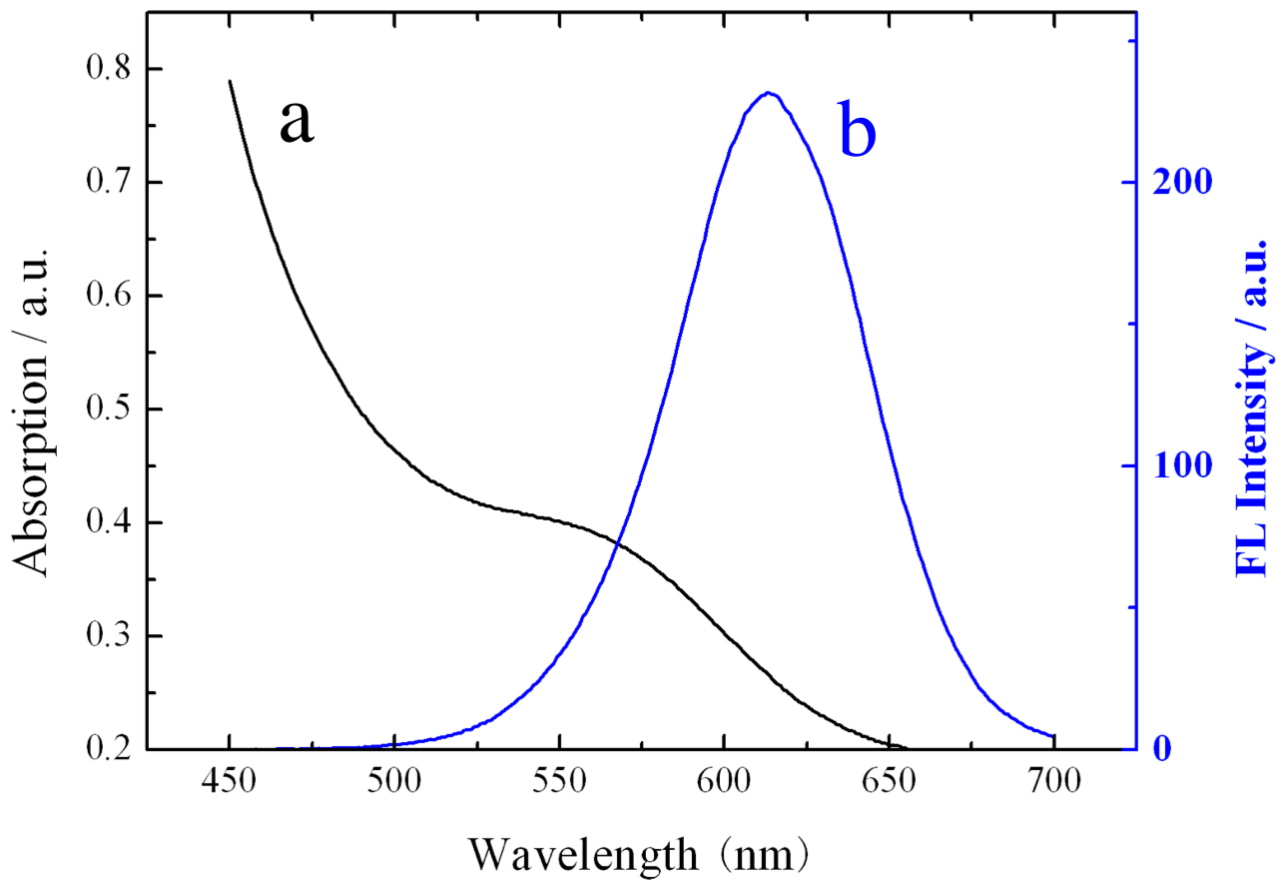
Absorption and fluorescence spectra of thiol-capped CdTe QDs in aqueous solution.

Water-soluble CdTe QDs were synthesized using the traditional one-pot approach. The UV-Vis absorption and fluorescence spectra of thioglycolic acid (TGA)-capped CdTe QDs are presented in Figure 2. The UV-Vis spectrum (Figure 2-a) indicates that the absorbance maxima of our TGA-capped CdTe QDs centered around 560 nm. The maximum emission was observed at 613 nm (Figure 2-b). The symmetric and narrow fluorescence peak indicated that the TGA-capped CdTe QDs were monodispersed.

The particle size can be calculated by the following empirical formula:

D = (9.8127×10^−7^) λ^3^−(1.7147×10^−3^) λ^3^+(1.0064) λ−194.84

In this formula, D (nm) is the particle diameter of the CdTe QDs, and λ (nm) is the wavelength of the first excitation absorption peak of the corresponding sample [17]. The particle size of the CdTe QDs was calculated to be approximately 3.5 nm.

The molar concentration was then calculated from the following equation [18]:

*C* = *A*/(10043*d* ^2.12^ *L*).

In this formula, *d* is the size of a given nanocrystal sample (nm), *A* is the absorbance of the excitation absorption peak of the corresponding sample, *C* is the molar concentration (M) of the QD solution, and *L* is the path length (cm) of the beam used for recording the absorption spectrum. In this work, *L* was fixed at 1.0 cm.

### Determination of the stability of CdTe QDs via fluorescence spectroscopy

**Figure 3:**
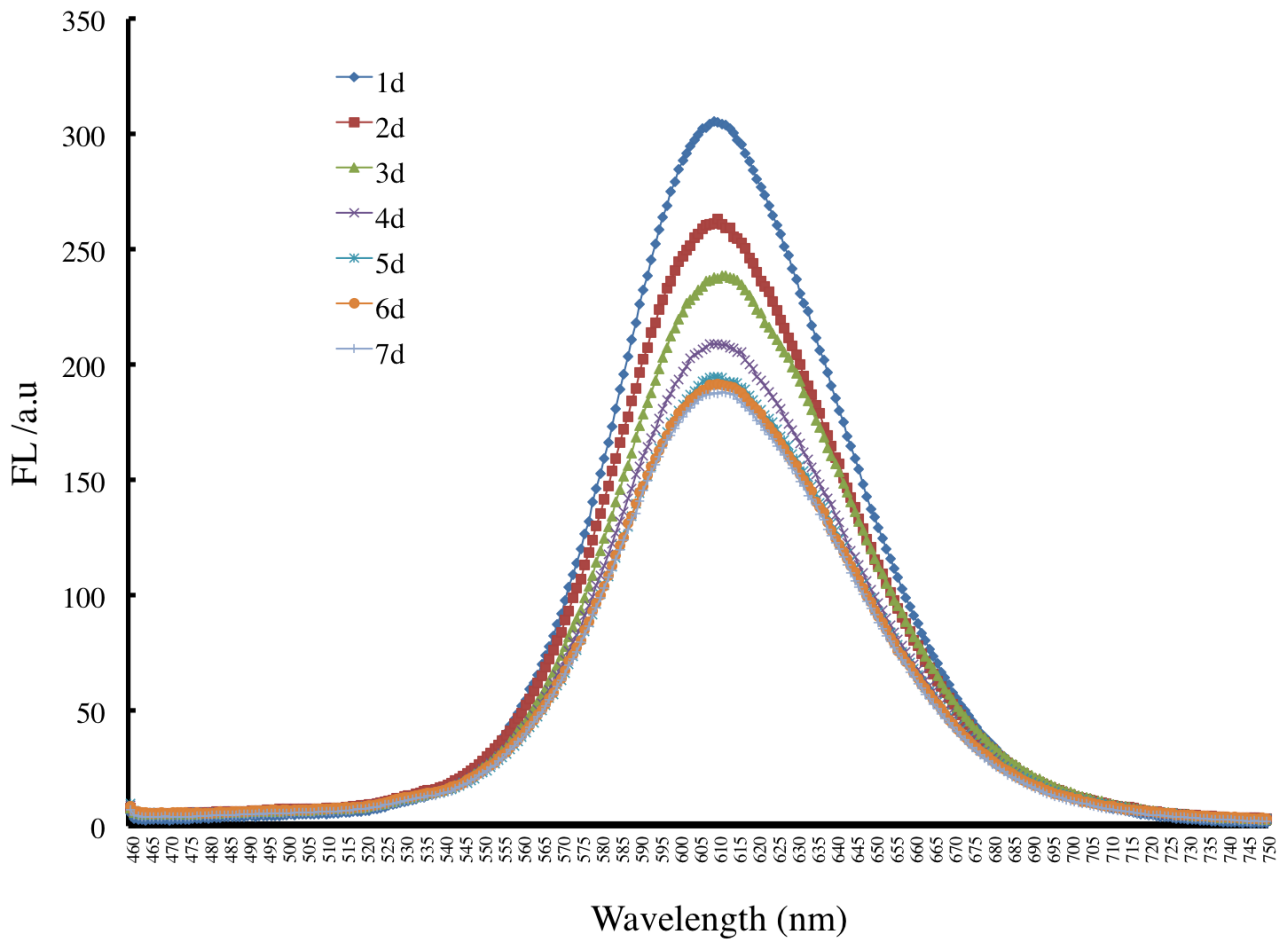
Time course of full wavelength scanning of TGA-capped CdTe QDs.

To test the stability of TGA-capped CdTe QDs, full wavelength scanning was conducted every day for 7 days. The results show that the fluorescence intensity of CdTe QDs varied over 7 days (Figure 3). The fluorescence intensity decreased monotonically during the first 4 days and reached a minimum that remained stable for the next 3 days (Figure 3).

### Cytotoxicity evaluation of CdTe QDs

Next, we evaluated the cytotoxicity of CdTe QDs on tobacco BY-2 cells by examining membrane permeabilization, intracellular reactive oxygen species production, cell death, and *phytochelatin synthase* (*PCS*) gene expression. The results demonstrated that the cytotoxicity of QDs is related to their concentration and the length of exposure time (Figure S1–Figure S5).

### CLV3 dodecapeptide modification eliminated the toxicity of CdTe QDs

Surface modification of QDs, including by peptide conjugation, is a common strategy for reducing their toxicity [28]–[30]. As a focus of this work, we studied the active CLE motif of CLV3, a secreted twelve-amino acid peptide hormone that targets receptors on plant stem cells (in the RAM and SAM) to regulate the proliferation and maintenance of stem cells in the meristematic zone [31]. To examine CLV3 conjugated to QDs, we first synthesized peptides using a peptide synthesizer and the Fmoc (9-fluorenylmethyloxy-carbonyl) solid-phase peptide synthesis method. The molecular mass of the synthesized peptides was confirmed by liquid chromatography-mass spectrometry (LC-MS) (Figure S6).

**Figure 4:**
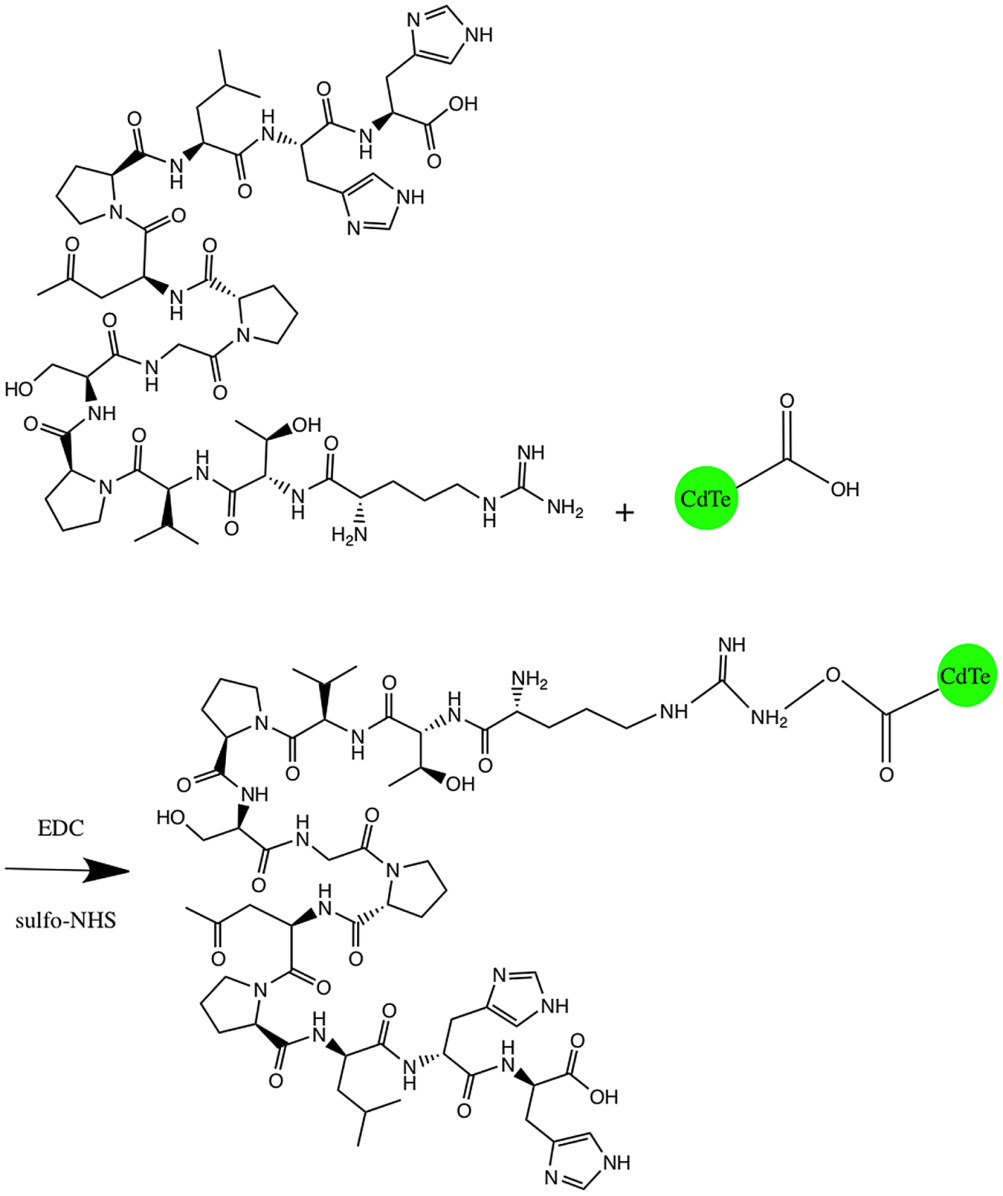
Zero-coupling procedure for conjunction of the CLAVATA3 (CLV3) dodecapeptide and CdTe QDs.

**Figure 5:**
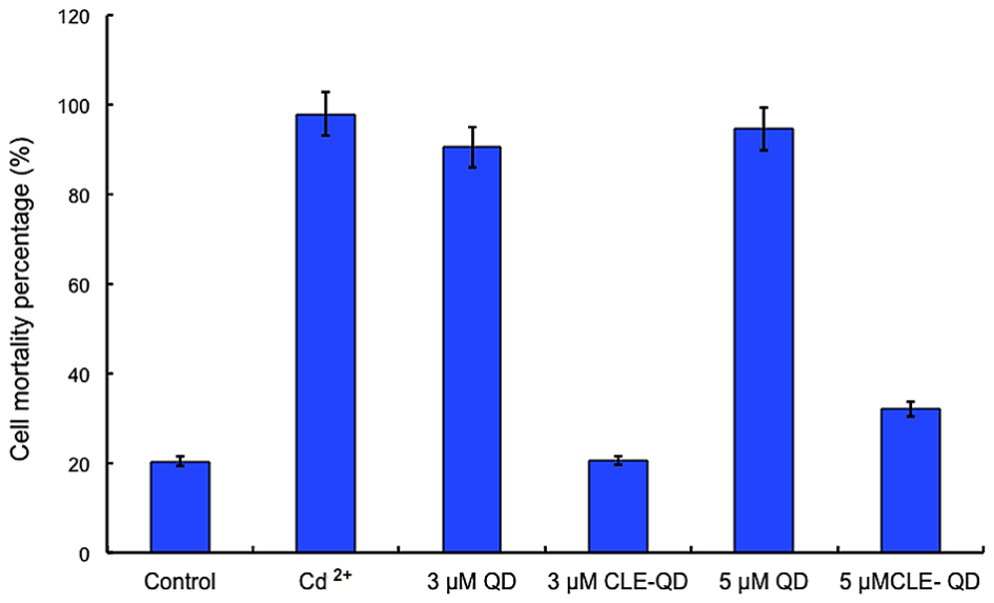
Peptide modification reduces the cytotoxicity of QDs. Data are the means of three independent replicates ± Standard Error (SE).

Next, we conjugated the dodecapeptide to the QDs by a zero-coupling procedure (Figure 4). We used agarose gel electrophoresis to confirm that the peptide and the QDs had linked, as the peptide-coated CdTe QDs showed reduced mobility compared to the uncoated QDs (Figure S7). After that, we assayed the cytotoxicity of the peptide-modified QDs. When compared with the same concentration of unmodified QDs, the peptide-coated QDs produced a much lower cell death rate (Figure 5). This result clearly indicated that this modification effectively reduced the toxicity of CdTe QDs in our experimental system.

### Root meristem stem cells can be labeled by CLV3-QD probes

**Figure 6:**
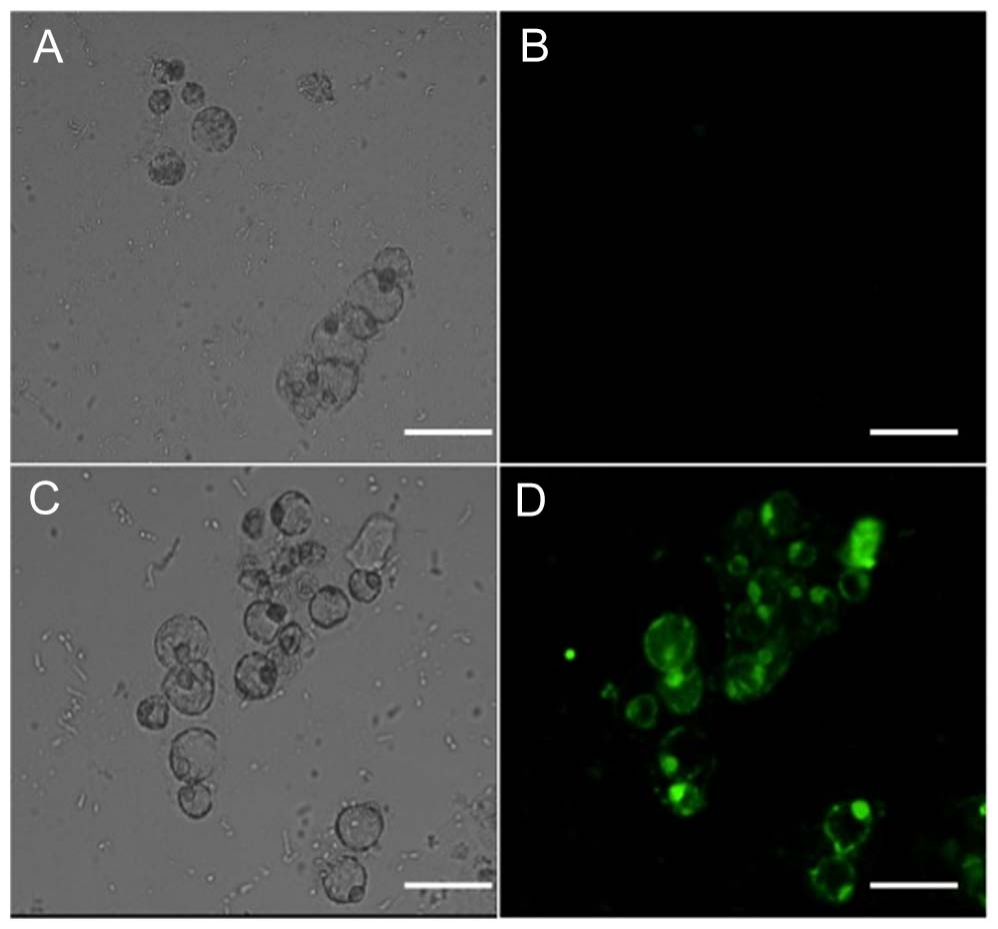
CLV3-QD localizes to the cell membrane of *Arabidopsis* root protoplasts. A. Bright field of control cells with QDs; B. fluorescent field of control cells incubated with QDs. After incubation, the probes were washed and no fluorescence was observed; C. Bright field of cells incubated with CLV3-QD probes; D. fluorescent field of C; after incubation, the probes were washed and a green fluorescent circle was observed at the membrane of the cells. Scale bar, 50 µm.

Ligand-receptor interactions are specific, and conjugation may alter the ligand conformation, potentially diminishing ligand-receptor affinity. The CLV3-QD probe is predicted to bind to peptide receptors on the membranes of stem cells. Accordingly, we examined the subcellular localization of CLV3-QDs in protoplasts derived from root stem cells. We incubated the probes (QDs with or without peptide modification) with the protoplasts generated from stem cells for 3 h and observed where the QDs localized. The cells were carefully washed 3 times with protoplast medium after incubation of the probes with the protoplasts to remove non-specific signal of probes. As a control, the unmodified QDs did not label the membrane (Figure 6A–B). After incubation and washing off excess CLV3-QD probes, we observed a green fluorescent circle clearly labeling the membrane of each cell (Figure 6C–D). These results indicated that peptide-modified probes specifically label the receptors on the plant stem cell membrane. The asymmetric fluorescence on the membrane indicated the asymmetric distribution of receptors on the membrane, which is of significance for the developmental fate of stem cells.

## Discussion

Ligand-receptor binding is a fundamental mechanism by which cells respond to their external environment to trigger changes in cell metabolism, membrane potentials, and transcription. In this paper, we synthesized CdTe nanoparticles, or quantum dots (QDs), and linked the CLV3 dodecapeptide to the surface of the CdTe QDs. We evaluated the bioactivity of this probe by examining its effects on plant root stem cells. Our results showed these dedecapeptide-modified QDs can be used as promising ligand probes to track plant stem cell fate at the optimized concentration.

### Nanoparticle size can be controlled by synthetic strategy

The advantage of a one-pot synthesis method is that the size of the nanoparticles can be easily controlled by regulation of the reaction temperature and time. Capping the QD surface with thiols is a basic approach for the conversion from hydrophobic QDs to hydrophilic QDs for biocompatible applications, which require further tailoring of the functional groups. The pioneering conversion of CdSe/ZnS QDs from the organic to the aqueous phase by surface capping using thioglycolic acid (TGA) was accomplished by Chan et al. and Bruchez et al [32], [33]. Subsequently, thiols were applied for direct synthesis of water-soluble QDs. For example, Gaoponik et al. synthesized water-soluble CdTe QDs by passing H_2_Te gas into cadmium thiolates that were prepared by stabilizing CdClO_4_⋅6H_2_O with thiols such as 2-mercaptoethanol (ME), thioglycerol (TG), TGA, and 2-(dimethylamino) ethanethiol (DAE)[34]. Various protocols were used for the synthesis of thiol-capped CdTe QDs [35]. In this paper, in contrast to our previous method to synthesize core-shell QDs [24], we synthesized TGA capped CdTe QDs, which were approximately 3.5 nm in diameter and showed strong band-edge emission.

### BY-2 cells were used to assess the cytotoxicity of the QDs

One dilemma in designing QDs is that decreased colloidal stability and increased nonspecific interactions often accompany a decrease in size. The potential cytotoxicity of nanoparticles in applications is related to their stability, as their degradation can release Cd^2+^, a common environmental and cellular heavy metal pollutant, which is not only toxic but also difficult to eliminate. The absorption of Cd^2+^ by cells can produce many toxic responses, alter cell morphology and organelle function, and eventually lead to cell death [36]. In this investigation, we determined the effect of CdTe QDs on cell membrane permeability. Our results indicated that QDs could not diffuse into BY-2 cells at the early incubation time (<2 d). With a longer incubation, we observed a fluorescent signal in the cytoplasm and nucleus (Figure S2), showing that QDs were present inside the cells. Consistent with the dye labeling results, this internalization may be related to the loss of cell membrane integrity (Figure S1, Figure S2 and S4). The mechanism for this membrane permeabilization is not yet clear. However, from our experiments on *PCS* gene expression, it is reasonable to hypothesize that Cd^2+^ could be released from the decomposition of CdTe QDs at long incubation time (>3 d). This released Cd^2+^ may be taken up by the cell and increase expression of *PCS*, whose protein product chelates Cd^2+^ to reduce its toxicity (Figure S5). However, with a much longer incubation time (5 d), *PCS* expression increased, and BY-2 cell death also increased, which was most likely caused by Cd^2+^. This was supported by an increase in cell mortality with the continuation of treatment.

As potential photosensitizers, QDs can transfer energy to another molecule. When the final acceptor molecule is water or oxygen, this leads to the generation of reactive oxygen species (ROS), which are lethal to bacteria, fungi, and mammalian cells. Previous studies have indicated that unmodified QDs could induce cell death through apoptosis. Considering this fact, much attention was paid to the modification of the surfaces of QDs to reduce their toxicity. Modification with carbohydrates, biotin or peptides can effectively reduce the cytotoxicity of QDs [15], [16], [37], [38]. In this research, we used another fluorescent dye to monitor ROS production in BY-2 cells under different treatment conditions. Our results demonstrated that treatment with QDs could enhance the production of ROS in cells, and the relative concentration of ROS increased over time during continuous treatment (Figure S3). The mechanism of ROS production is not clear. However, it is most likely an effect of the destruction of the membranes by QDs after long exposure time.

### CLAVATA3 (CLV3) dodecapeptide modification of the CdTe QDs can be used as promising probe for plant stem cell imaging

Unmodified CdTe QDs are less accessible to biomolecules, more likely to release toxic ions, and susceptible to photobleaching; thus their use in biological imaging has been limited. The modification of biocompatible QDs to reduce their toxicity is a prerequisite for cell labeling studies. Multiple hydrophilic chemical groups can be added to the QD surface to stabilize the QDs and reduce their toxicity. However, the selection of the surface modification groups requires investigation, and depends on the particular application, such as extracellular labeling, intracellular delivery, intracellular labeling, or *in vivo* imaging. In this investigation, we tethered a small peptide onto the surface of CdTe QDs (Figure 4;Figure S7). Our results demonstrated that this modification greatly decreased the toxicity of QDs to BY-2 cells (Figure 5) and that these modified QDs are a promising probe for further investigations.

The presentation of chemical information at the same size scale as that of cell surface receptors could potentially serve as a powerful means for understanding the interaction of ligands with cell receptors and directing cell function. However, the conjugation of a ligand to the QD surface will most likely significantly inhibit receptor binding due to the steric hindrance of the bulky QDs. Therefore, it is important to determine the bioactivity of peptide-capped QDs. In this research, we linked a plant peptide hormone to the CdTe QDs, and successfully monitored their *in vivo* biological labeling of Arabidopsis stem cells (Figure 6).

**Figure 7:**
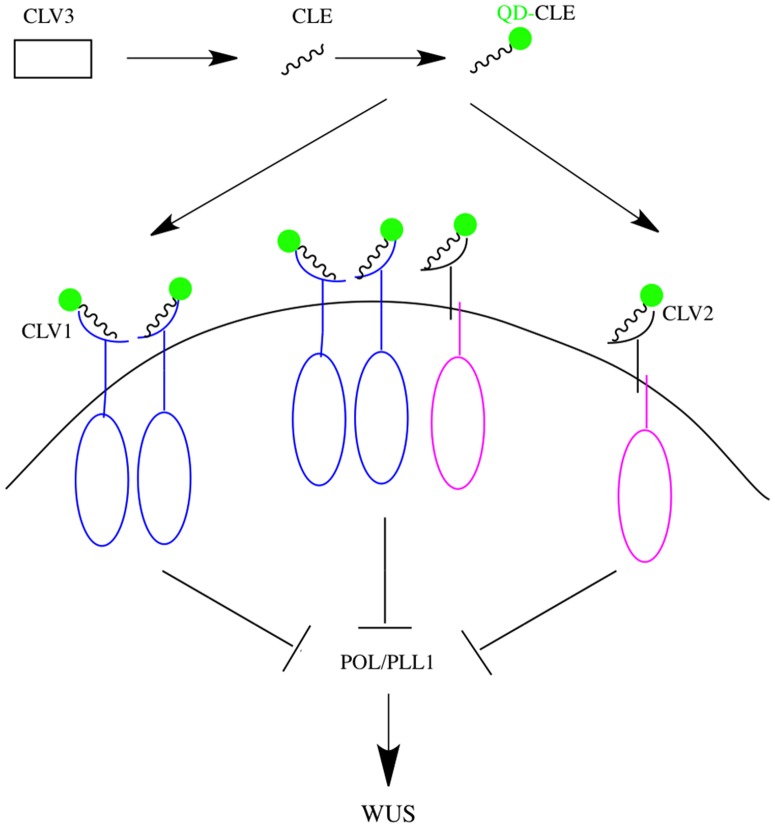
A model for CLV signaling (Adapted from [46] and [47]).

Presently, although other kinds of nanoparticles, such as gold nanoparticles [39], [40], silver nanoparticles [41] and NaYF_4_∶Yb [42] have been developed and used for bioimaging, current technology has remained at the stage of synthesis and physical-chemical characterization, and mainly used for gene delivery and fluorescent detection [39]–[42]. For the CdTe QDs, some reports showed that capping with silica or polymer [43]–[45] can stable the QDs; however, the rigid chemical structure greatly hindered their applications in biological *in vivo* imaging, and they have limited uses for molecular imprinting of proteins, or fluorescent detection of toxic organic solvents [43]–[45]. Here, to gain insight into the role of the CLV3 peptides in cell-cell reciprocal communication, we synthesized CdTe QDs via the one-pot method, and then conjugated synthetic CLV3 peptides to the surface of the QDs. This modification reduced the toxicity of QDs to plant cells. Therefore, these results provide promising probes for plant stem cell labeling and cell fate tracking (Figure 7). In this model, CLE signaling peptides arising from CLV3 are proteolytically processed and labeled by QDs. These modified functional QDs can be used as the active probes to bind both a CLV1 homodimer and the CLV2-CRN (CORYNE) complex. These two activated receptor complexes either interact with each other or signal independently to repress phosphatase type 2C proteins, POL (POLTERGEIST)-PLL1 (POL-Like 1) activity, and at last activiate WUSCHEL (WUS) to determine the fate of stem cells.

## Supporting Information

Figure S1 **Cell damage assay using SYTOX green uptake for different treatment conditions.** Scale bar, 50 µm.
(TIF)

Figure S2 **Concentration-dependent diffusion of CdTe QDs into BY-2 cells. The left panel is the bright field of BY-2 cells, and the right panel is the fluorescence at each concentration.** The images were captured at each time point using fluorescence microscopy. Scale bar, 50 µm.
(TIF)

Figure S3 **Reactive oxygen species (ROS) production with different treatments.** Scale bar, 50 µm.
(TIF)

Figure S4 **Cell death assay with Evans Blue.** Images were captured using bright field microscopy. Scale bar, 50 µm.A–D. Cell death assay with Evans Blue at 1 d of treatment. A. Control, B. 1.0 µM Cd^2+^ treatment, C. 1.0 µM CdTe QD treatment, D. 5.0 µM CdTe QD treatment. E–H. Cell death assay with Evans blue at 5 d of treatment. E. Control, F. 1.0 µM Cd^2+^ treatment, G. 1.0 µM CdTe QD treatment, H. 5.0 µM CdTe QD treatment. I. This graph shows the statistical data of cell death in different treatments at different time. Values represent the mean ± Standard Error (SE) from three independent experiments.
(TIFF)

Figure S5 **Relative expression of** ***phytochelatin synthesis (PCS)*** **in different treatments.** *Actin* was used as the internal control. Data are the means of three independent replicates ± Standard Error (SE).
(TIF)

Figure S6 **The synthesized dodecapeptide by Fmoc (9-fluorenylmethyloxy-carbonyl) solid-phase peptide synthesis method and liquid chromatography-mass spectrometry (LC-MS) analysis.** Insert panel: structure of the synthesized dodecapeptide.
(TIF)

Figure S7 **Mobility shift was assayed by 0.5% agarose gel electrophoresis.** The mobility of the peptide-coated QDs (left band) lagged behind the uncoated QDs (control) (right band).
(TIF)

Data S1 **Supplementary results, figures, and references.**
(ZIP)
