# Supplementary figures and images for: CLAVATA3 Dodecapeptide Modified CdTe Nanoparticles: A Biocompatible Quantum Dot Probe for In Vivo Labeling of Plant Stem Cells
Source: PLoS One. 2014 Feb 24;9(2):e89241. doi: 10.1371/journal.pone.0089241 (PMC3933426; doi:10.1371/journal.pone.0089241)

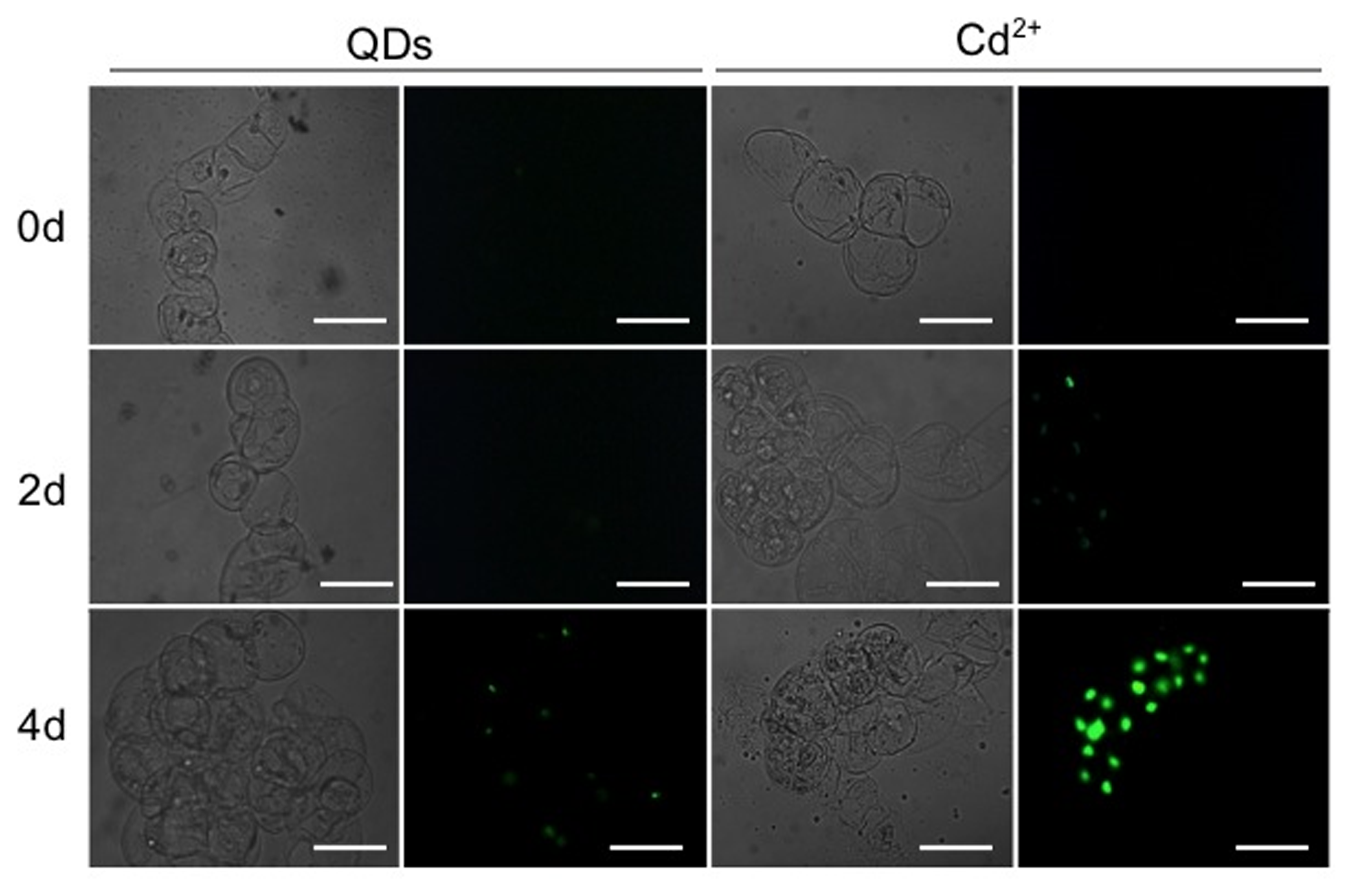

Supplement: Figure S1 — Cell damage assay using SYTOX green uptake for different treatment conditions. Scale bar, 50 µm. (TIF) [file pone.0089241.s001.tif]

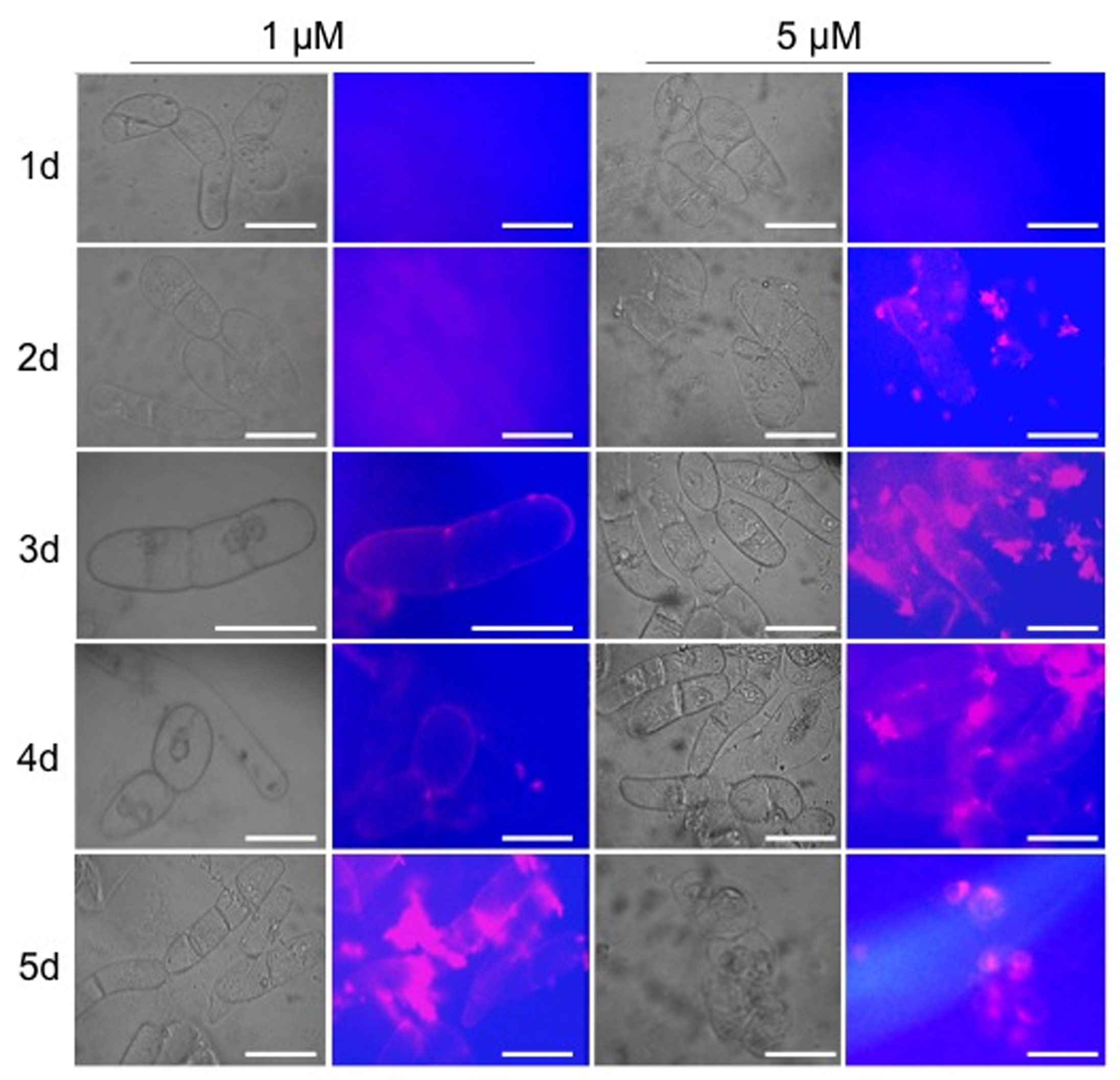

Supplement: Figure S2 — Concentration-dependent diffusion of CdTe QDs into BY-2 cells. The left panel is the bright field of BY-2 cells, and the right panel is the fluorescence at each concentration. The images were captured at each time point using fluorescence microscopy. Scale bar, 50 µm. (TIF) [file pone.0089241.s002.tif]

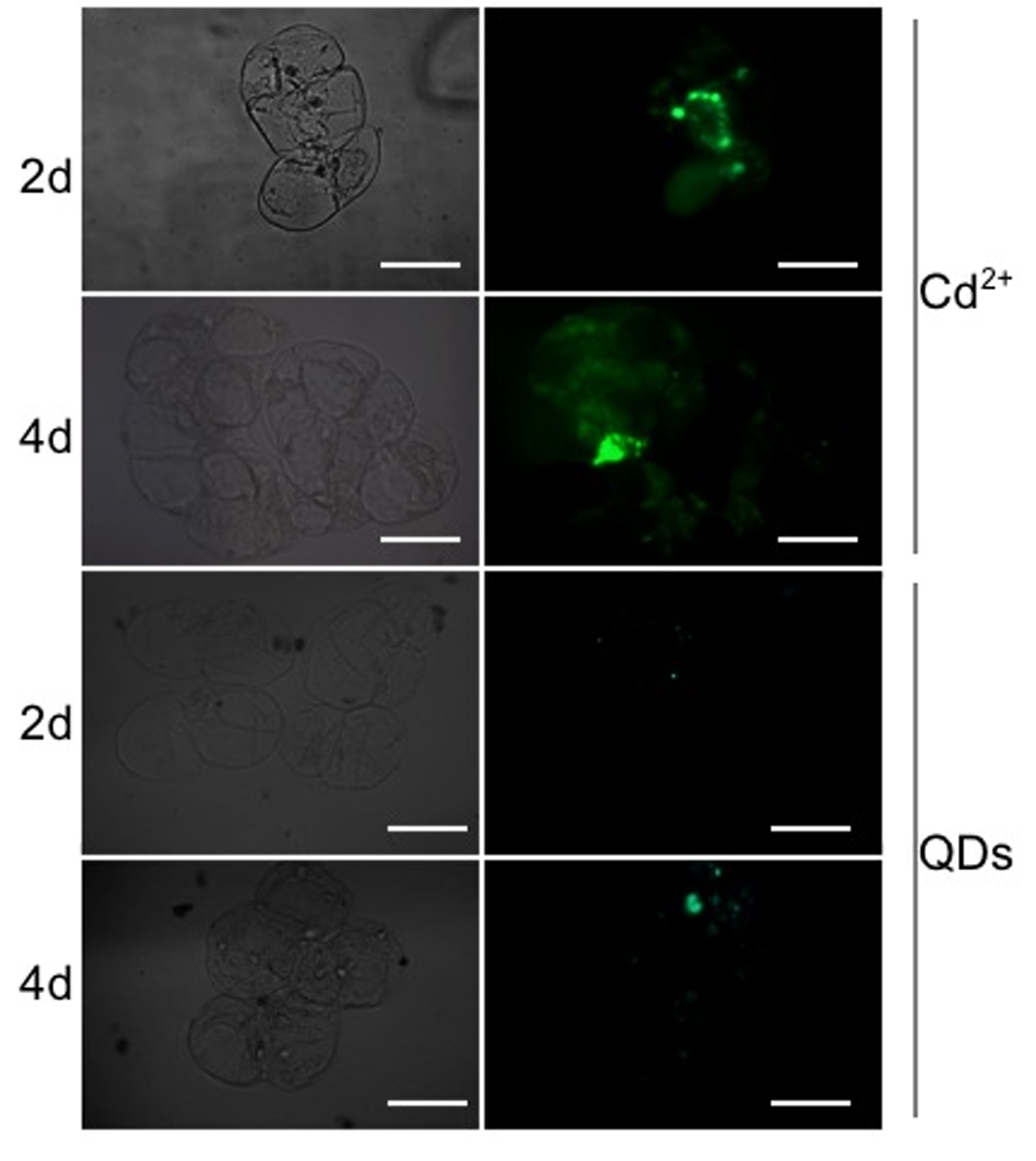

Supplement: Figure S3 — Reactive oxygen species (ROS) production with different treatments. Scale bar, 50 µm. (TIF) [file pone.0089241.s003.tif]

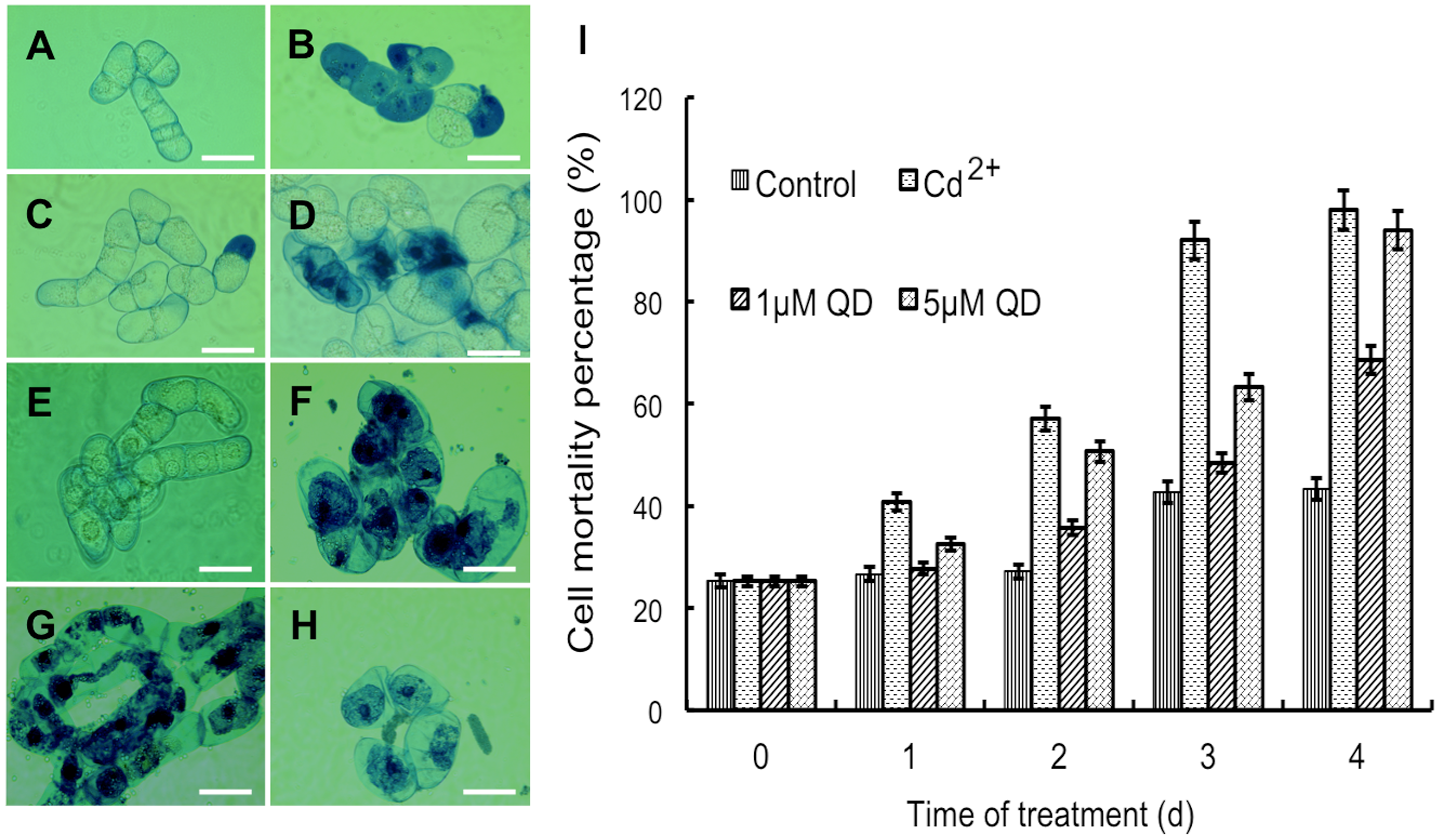

Supplement: Figure S4 — Cell death assay with Evans Blue. Images were captured using bright field microscopy. Scale bar, 50 µm.A–D. Cell death assay with Evans Blue at 1 d of treatment. A. Control, B. 1.0 µM Cd2+ treatment, C. 1.0 µM CdTe QD treatment, D. 5.0 µM CdTe QD treatment. E–H. Cell death assay with Evans blue at 5 d of treatment. E. Control, F. 1.0 µM Cd2+ treatment, G. 1.0 µM CdTe QD treatment, H. 5.0 µM CdTe QD treatment. I. This graph shows the statistical data of cell death in different treatments at different time. Values represent the mean ± Standard Error (SE) from three independent experiments. (TIFF) [file pone.0089241.s004.tiff]

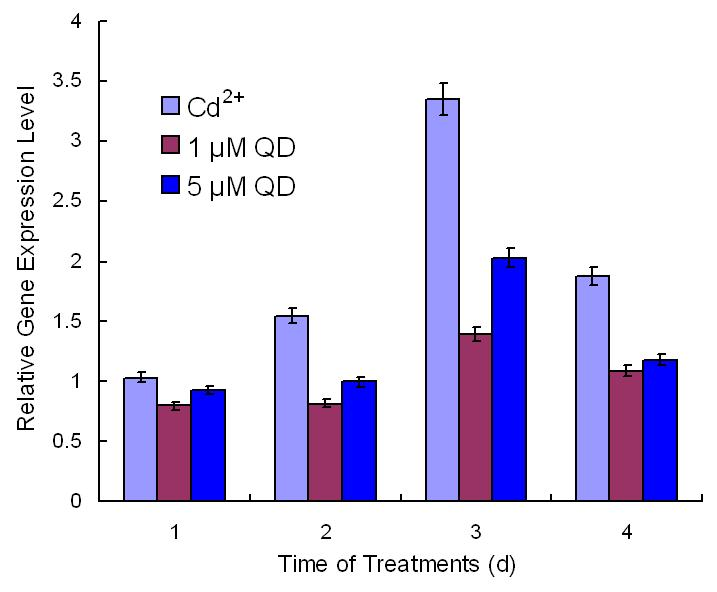

Supplement: Figure S5 — Relative expression of phytochelatin synthesis (PCS) in different treatments. Actin was used as the internal control. Data are the means of three independent replicates ± Standard Error (SE). (TIF) [file pone.0089241.s005.tif]

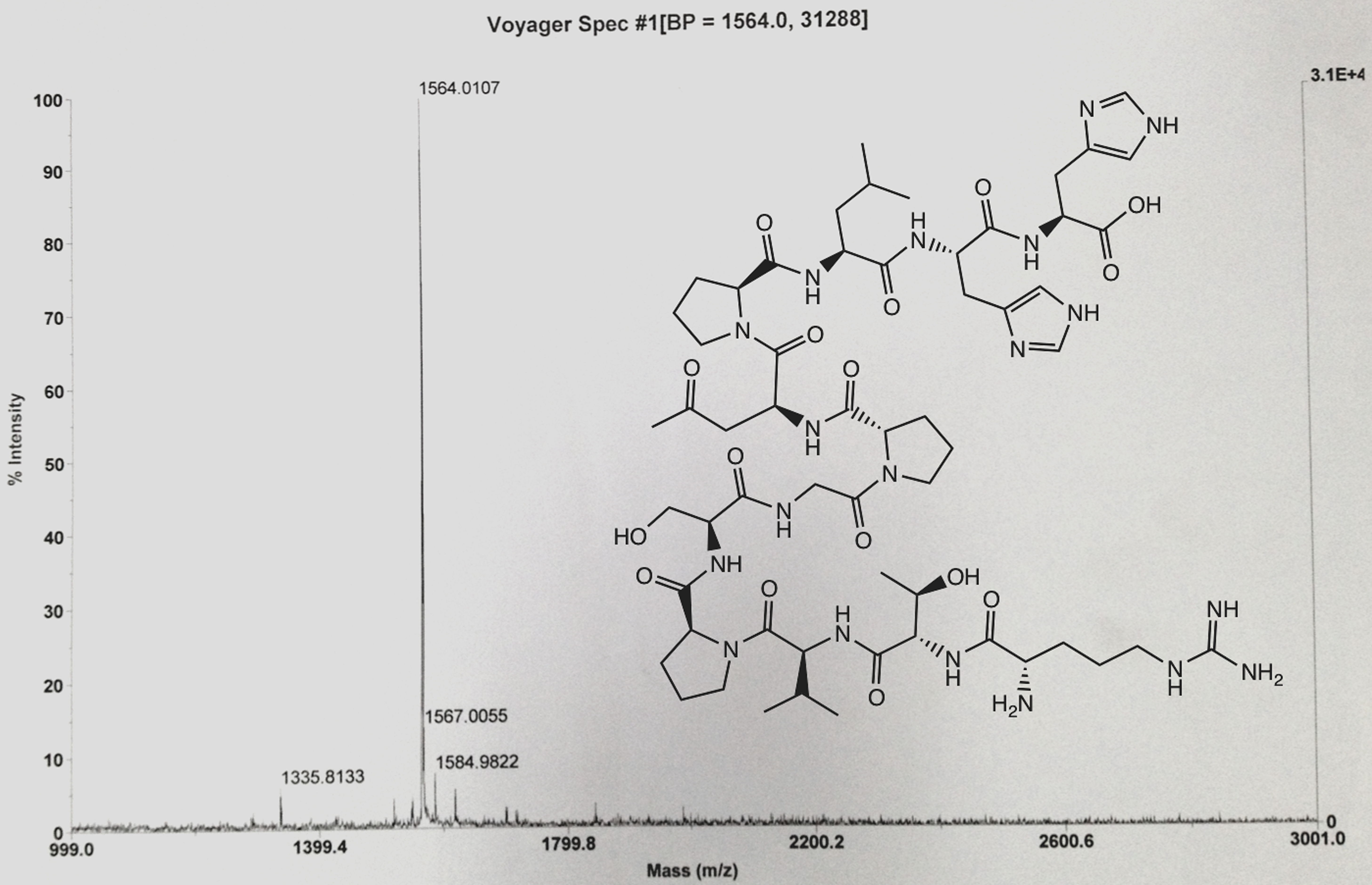

Supplement: Figure S6 — The synthesized dodecapeptide by Fmoc (9-fluorenylmethyloxy-carbonyl) solid-phase peptide synthesis method and liquid chromatography-mass spectrometry (LC-MS) analysis. Insert panel: structure of the synthesized dodecapeptide. (TIF) [file pone.0089241.s006.tif]

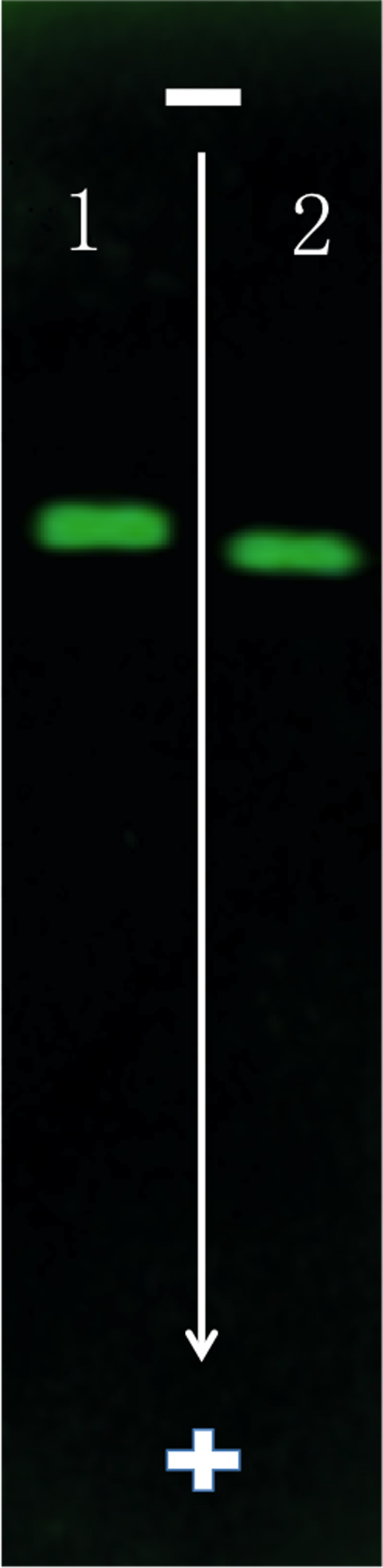

Supplement: Figure S7 — Mobility shift was assayed by 0.5% agarose gel electrophoresis. The mobility of the peptide-coated QDs (left band) lagged behind the uncoated QDs (control) (right band). (TIF) [file pone.0089241.s007.tif]
